# Editorial: CRISPR-Cas in Agriculture: Opportunities and Challenges

**DOI:** 10.3389/fpls.2021.672329

**Published:** 2021-03-26

**Authors:** Sandeep Kumar, Linda Ann Rymarquis, Hiroshi Ezura, Vladimir Nekrasov

**Affiliations:** ^1^Corteva Agriscience, Johnston, IA, United States; ^2^Bayer Crop Science, Chesterfield, MO, United States; ^3^Faculty of Life and Environmental Sciences, University of Tsukuba, Tsukuba, Japan; ^4^Tsukuba Plant Innovation Research Center (T-PIRC), University of Tsukuba, Tsukuba, Japan; ^5^Plant Sciences Department, Rothamsted Research, Harpenden, United Kingdom

**Keywords:** agriculture, genome editing, crop, CRISPR, Cas9, Cas12a, policy, regulation

## CRISPR-CAS Technology: State of the Art, Policy, and Regulation

CRISPR-Cas genome editing technology is developing at a rapid pace and new molecular tools, such as CRISPR nucleases, are becoming regularly available. As part of this Research Topic, Bandyopadhyay et al. provide a comprehensive overview of Cas12a, a CRISPR nuclease formerly known as Cpf1. In their review article, the authors cover structural and mechanistic aspects of Cas12a in comparison with Cas9, the most commonly used CRISPR nuclease. They also highlight uses of Cas12a for the purpose of improving agriculturally important traits in various crops. An overview of Cas9 genome editing applications in plants is provided by El-Mounadi et al. who introduce the reader to the mechanism of Cas9 activity, methods of its delivery to plant cells (i.e., transformation techniques), give examples of improving crop traits using CRISPR-Cas9, and touch on biosafety and regulatory aspects associated with genome editing. A number of countries (e.g., the USA, Brazil, Argentina, and Japan) have already exempted genome edited crops, which do not carry transgenic DNA or novel combination of genetic material (i.e., not similarly achievable through conventional breeding), from being regulated similarly to Genetically Modified Organisms (GMOs) as genetically engineered (GE) organisms (Schmidt et al., 2020). Although the above-mentioned countries have passed legislation allowing cultivation of genome edited crops without GE regulation, the public dialogue and policy developments on the issue are evolving. In the case of Japan, Tabei et al. analyze Twitter conversation on genome-edited foods and their labeling over the period from May to October 2019. The analysis reveals that 54.5% of relevant tweets were statements opposed to food produced using genome edited crops, while only 7% were statements in favor of it. The remaining 38.5% of tweets were statements deemed neutral. Although the analysis was not necessarily representative of the wider Japanese society due to bias among Twitter users, the study underlines the importance of a continuous public dialogue on the issue of genome edited crops in Japan and the rest of the world.

## CRISPR-Cas as a Tool for Gene Function Studies and Crop Trait Improvement

One of the factors impacting the efficiency of CRISPR-Cas is the expression level of the gene encoding the respective nuclease during different developmental stages of the plant. For example, CRISPR-Cas mutagenesis in Arabidopsis often results in chimerism in the T1 generation due to low expression of *Cas9* (when driven by a promoter, such as 35S) during the zygote and early embryo developmental stages (Feng et al., 2014). To address the chimerism problem, egg cell-specific promoters could be a good choice to drive CRISPR nuclease gene expression for increasing the rates of CRISPR-Cas-induced germline mutations, which are inherited by the next generation (Wang et al., 2015; Yan et al., 2015; Mao et al., 2016). Zheng et al. test four different egg cell-specific promoters (two from Arabidopsis and two from soybean) to drive expression of *Cas9* in Arabidopsis and soybean transgenic lines. Out of the four promoters, AtEC1.2e1.1p, which is an adaptation of the previously published *AtEC1.1* and *AtEC1.2* promoter fusion (Wang et al., 2015), seems to perform best in both plant species. The study by Zheng et al. therefore contributes new molecular tools for efficient targeted mutagenesis in a model plant, such as Arabidopsis, and an economically important crop, such as soybean.

Virdi et al. and Zhang et al. highlight the use the CRISPR-Cas for gene function analysis. Virdi et al. studied the soybean *KASI* gene, which is crucial for conversion of sucrose to oil. They demonstrate that CRISPR-Cas induced knockout and in-frame deletion of *GmkasI* alleles, have an increase in seed sucrose content and a decrease in total seed oil content relative to wild type. These phenotypes are consistent with what was observed in the mutant line where the *GmKASI* gene is disrupted by a reciprocal chromosomal translocation. Thus, the authors prove that the phenotype of the line carrying the chromosomal translocation is indeed due to disruption of the *GmKASI* gene.

Zhang et al. report on knocking out 63 genes involved in immune response in tomato. The authors performed a detailed analysis of the types of mutations generated at an average frequency of 68%, which is similar to previously reported rates for CRISPR-Cas mutagenesis in tomato (Brooks et al., 2014; Nekrasov et al., 2017). They demonstrate that the mutations were transmitted through the germline to the next generation. The off-target analysis they performed for 12 guide RNAs showed no mutations at off-target sites with up to four mismatches and, indicating the high precision of CRISPR-Cas in tomato, this was consistent with what was previously reported (Nekrasov et al., 2017; Hahn and Nekrasov, 2019). The knockout lines are cataloged in the online Plant Genome Editing Database (PGED; http://plantcrispr.org; Zheng et al., 2019).

The CRISPR-Cas technology is a versatile genome editing tool that has been used to improve agriculturally important crop traits, such as quality, disease resistance, and herbicide tolerance. In potato, enzymatic browning is a serious problem for both growers and the industry as it decreases the quality of both the fresh and processed product. González et al. report on a successful application of CRISPR ribonucleoproteins for the purpose of reducing enzymatic browning in potato tubers by targeting the Polyphenol Oxidase 2 gene (*StPPO2*), one of the five potato PPO genes. By disrupting all four copies of *StPPO2* the authors achieved a dramatic reduction in tuber PPO activity (up to 69%) and enzymatic browning (73%). The findings presented by González et al. are consistent with the reported reduction in potato browning achieved by silencing the *StPPO2* gene using RNAi (Richael, 2021).

In addition to dicot crops, CRISPR-Cas has been extensively used for trait improvement in cereals, such as rice and maize. As an example, Zafar et al. are reporting on enhancing disease resistance to *Xanthomonas oryzae* pv. *oryzae* (*Xoo*), a pathogen causing bacterial blight in rice, by editing the promoter of a susceptibility (S) gene. *Xoo* secretes transcription activator-like effectors (TALEs) that activate host S genes, such as Os*SWEET* family members. Using CRISPR-Cas, the authors introduced deletions overlapping with effector binding elements (EBEs) recognized by AvrXa7/PthXo3 or TalF TALEs within the promoter of the *OsSWEET14* gene in the Super Basmati elite cultivar. Mutant rice lines carrying deletions in the AvrXa7/PthXo3 EBEs showed enhanced resistance to the *Xoo* strain carrying *AvrXa7* in agreement with previously published reports (Li et al., 2012; Blanvillain-Baufumé et al., 2017; Oliva et al., 2019; Xu et al., 2019).

In another report, Komatsu et al. address the problem of “volunteer rice” that emerges from seeds falling into fields during the harvest season and then spontaneously germinates the next spring. If volunteer rice originates from a feed variety, it can compromise the quality of rice meant for human consumption, which is grown during the next season. As many japonica rice cultivars are resistant to beta-triketone herbicides (bTH), such as benzobicyclon (BBC), the authors tested the feasibility of engineering BBC susceptibility in japonica rice (cv. Nipponbare) by targeting the *HIS1* gene using the cytosine base editor (CBE). They successfully generated a number of *his1* knockout lines by eliminating the start codon or introducing premature stop codons within the *HIS1* coding sequence. The *his1* loss-of-function lines appear to be susceptible to BBC and other beta-triketone pesticides, paving a way to controlling volunteer rice in the field by applying the same strategy to BBC-resistant feed rice cultivars.

In maize, Gao et al. report on a CRISPR-Cas9- and recombinase-mediated strategy for stacking biotech traits within complex trait loci (CTLs). Each CTL spans 4–5 cM and includes 12–30 pre-selected sites used for insertion of a landing pad via homology-directed repair (HDR) using CRISPR-Cas9. As a result, the authors generated a set of individual transgenic lines, each carrying a landing pad at one of the preselected sites within one of the four CTLs. At the following step, the landing pad-carrying lines were used for integration of trait genes using the FLP recombinase. Finally, integrated trait genes were stacked on the same chromosome by crossing respective individual transgenic lines and selecting recombinants. The study by Gao et al. therefore presents a modular and flexible way of stacking biotech traits, as compared to previously reported strategies involving recombinases or zinc finger/homing endonucleases (Ow, 2011; D'Halluin et al., 2013; Kumar et al., 2015), due to the possibility for trait genes to be easily combined or separated (e.g., in case one of them loses efficiency) by conventional breeding.

This volume highlights the many opportunities that the CRISPR-Cas systems hold for Agriculture. Both Cas9 and Cas12a have been proven to drive edits in plants and new improvements, such as using germline-specific promoters that increase heritability, will only enhance their potential (Zheng et al.). The CRISPR-Cas systems have been successfully implemented to forward basic research, such as gene discovery of oil (Virdi et al.) and disease genes (Zhang et al.), as well as improve agricultural outcomes e.g., via decreasing potato browning (González et al.), improving disease resistance (Zafar et al.), mitigating volunteer rice (Komatsu et al.), and stacking biotech traits (Gao et al.). In order for this potential to be fully realized, Tabei et al. showed that work must be done to gain public acceptance and ensure implementation of favorable public policy.

## Author Contributions

VN wrote the paper. SK, LR, and HE made direct contributions to the work, reviewed, and approved it for publication. All authors contributed to the article and approved the submitted version.

## Conflict of Interest

SK is an employee of Corteva Agriscience™. LR is an employee of Bayer. The remaining authors declare that the research was conducted in the absence of any commercial or financial relationships that could be construed as a potential conflict of interest.
